# Cross-reactivity in Grasses: Biochemical Attributes Define Exemplar Relevance

**DOI:** 10.1097/WOX.0b013e31826a10cf

**Published:** 2012-10-15

**Authors:** Alan Bullimore, Toby Batten, Simon Hewings, Karl Juergen Fischer von Weikersthal-Drachenberg, Murray Skinner

**Affiliations:** 1 Allergy Therapeutics, Dominion Way, Worthing, West Sussex, UK

**Keywords:** allergens, characterization, homology, grass, immunotherapy

## Abstract

**Introduction:**

Broad-spectrum grass pollen immunotherapies contain large numbers of allergenic proteins from multiple species. The principle of homologous grouping is used as a tool to assist in the standardization of allergen immunotherapy. This study reviews the principle of homologous grouping, questions what an exemplar grass should be, and queries whether a 1-way system of inferring homology is appropriate.

**Methods:**

Grass pollens were extracted and analyzed using a variety of techniques, including enzyme-linked immunosorbent assay, Bradford protein assay, sodium dodecyl sulfate-polyacrylamide gel electrophoresis, and quantitative analysis of Western blots.

**Results:**

Variation in protein content, IgG, IgE, and Phl p 5 reactivity is evident among all grasses analyzed. There is significant evidence of similarity but also disparity consistent with variation resulting from evolutionary change. Proprietary software called Gel Electrophoresis Protein Profile Analysis has been developed, which highlights that each grass exhibits a greater than 55% similarity measure; this is considered high similarity.

**Discussion:**

None of the grass species examined display an identical biological profile. However, data indicate that there is a high degree of homology, and Crested Dogstail is similar to each of the other 12 species analyzed; these levels of similarity can only be possible because of molecular profile and extensive sharing of epitopes. These data are considered to be sufficient to include Crested Dogstail within the sweet grasses group of the Pooideae family; however, the subtle differences in grasses also justify the inclusion of multiple species to create a broad-spectrum immunotherapy.

## 

Allergic sensitivity to grass pollen is responsible for 40% of allergic cases worldwide [1]. The group 1 and group 5 allergens are considered as major allergens due to 90% and 65 to 85% of grass pollen allergic individuals possessing IgE reactivity to each allergen, respectively [2]. However, individuals sensitized solely to minor allergens comprise a significant number of grass pollen allergic patients. One study found that 11% of patients were sensitized only to minor allergens [3], suggesting that although these allergens may be "minor", they are not "unimportant."

The symptoms of allergy are the result of histamine release, triggered by cross-linked allergen-specific IgE on the surface of mast cells and basophils, along with other inflammatory mediators, such as leukotrienes. The subsequent recruitment of eosinophils is completed by the action of T-cell cytokines, such as interleukin (IL)-4 and IL-5.

Specific immunotherapy (SIT) is recognized by the World Health Organization, aside from allergen avoidance, as the only treatment "that may affect the natural course of allergic diseases."[4] The mechanism through which SIT operates has yet to be fully understood; however, SIT is thought to function by the action of IL-12 inducing a shift from a T_H_2 response to a T_H_1 CD4^+ ^cytokine profile. IL-10 and Transforming Growth Factor (TGF)-β reduce the production of antigen-specific IgE and proinflammatory cytokine release [5-15].

Many immunotherapies are manufactured from natural raw materials. These extracts are comprised of many solubilized proteins, and because the use of multiple species has been linked to potential treatment benefit [16], multispecies therapies contain a large number of allergenic proteins. This multifarious nature invariably means that characterization is more complex than that for a single-species product.

Criticism of the capacity to sufficiently characterize and thus standardize a product [17] risks a movement to simplify allergen therapies, potentially leading to the exclusion of minor allergens and multiple grass species. This could lead to a potential reduction in patient benefit. Novel methods to characterize immunotherapies, including mass spectrometry, amino acid sequencing, and proteomics, are enabling complex allergen mixtures to be characterized in a way that was not possible before.

To facilitate allergenic species standardization, Lorenz et al [18] grouped species according to the principle of homologous groups and allowed provision for the inclusion of more species as further data became available. Recent findings have demonstrated that the grasses of the Pooideae group, although cross-reactive and homologous, contain varying amounts of major allergen [19]. Moingeon et al [19] conclude that this, coupled with the inclusion of multiple species, mimics more natural exposure conditions.

A broad-spectrum product comprising 13 different grass species such to better mimic the natural exposure profile alluded to by Moingeon et al (2008) has been investigated. This ultrashort course therapy is demonstrated to be well tolerated and suitable for the treatment of allergic rhinitis [20]. Twelve of the constituent grass species are recognized as homologous to Timothy grass (*Phleum pratense*) by Lorenz et al [18]. The thirteenth grass species--Crested Dogstail (*Cynosurus cristatus*)--was unable to be classified as homologous with the sweet grasses of the Poaceae family due to lack of information available to the authors at the time.

Consequently, this work investigated a 13-grass product mix to determine the allergenic and allergomic profile of the 13 component grass species using similarity assessments, epitope blocking, in vitro analysis, and proprietary pattern-matching algorithms. The data were used to conclude whether, according to the criteria stated by Lorenz et al [18], Crested Dogstail can be considered homologous to the sweet grasses group of the Pooideae family. This study additionally reviews the principle of homologous grouping, questions what an exemplar grass should be, and queries whether a 1-way system of inferring homology is appropriate.

## Materials and Methods

### Grass Pollen Extracts

Grass pollens were purchased from Allergon (Ängelholm, Sweden) and Pharmallerga (Lisov, Czech Republic). Five percent single-pollen extracts were prepared by roller mixing 2.31 g of pollen in 46.15 mL of phosphate extraction buffer (1 μM Na_2_HPO_4_, 271.89 nM KH_2_PO_4_, 8.56 μM NaCl, 0.5% vol/vol phenol, 2 M HCl, and 2 M NaOH) at 2 to 8°C for 18 hours. A 13-grass mix was prepared by roller mixing 2.31 g of each of the 13 grasses in 600 mL of phosphate extraction buffer at 2 to 8°C for 18 hours. This was followed by centrifugation at 3000*g *for 10 minutes to sediment the solid material. Subsequently, the supernatant was clarified by passage through a 0.2-μm syringe filter (Millipore, Watford, United Kingdom). The filtered extracts were then stored at 2 to 8°C until required for use.

### Serum Pool Creation

Three serum pools were constructed with serum sourced from PlasmaLabs (Everett, WA). The first pool was created with 8 grass-positive patients (1 female and 7 males, of whom 6 were of white and 2 were African American ethnicities). The second pool was created with 20 grasspositive patients (7 female and 13 males, of whom 13 were of white descent, 3 of Asian descent, 1 of Hispanic descent, and 1 of African American descent). The third pool was created with the intention of blocking reactivity to Timothy grass epitopes and composed of the 20-patient pool described above plus the addition of native Timothy grass extract prepared as above to a final concentration of 1:5.

### Sodium Dodecyl Sulfate-Polyacrylamide Gel Electrophoresis

Aliquots of the pollen extracts were denatured by heating at 100°C for 2 minutes in a sample buffer containing sodium dodecyl sulfate. The proteins were then resolved on a 10 to 20% Tris-HCl Criterion gel (Bio-Rad, Hemel Hempstead, United Kingdom) and the gels electrophoresed according to the manufacturer's protocol. The separated proteins were transferred onto polyvinyldifluoride membranes using a semidry apparatus (Bio-Rad). The membranes were then washed and Western blotted as described below.

### Western Blotting

A polyvinyldifluoride membrane was blocked with 10% milk diluent (KPL, Middlesex, United Kingdom) prepared in Dulbecco phosphate-buffered saline (DPBS). The membranes were then washed with phosphate-buffered saline-0.3% Tween 20 and incubated overnight at 4°C with the human patient grass-positive sera pools prepared as above. All IgE sera pools from grass allergic individuals were used at a 1:5 dilution (vol/vol in 5% milk diluent) and incubated overnight at 2 to 8°C. The membranes were washed again and incubated with 1 mg/mL of biotinylated goat anti-human IgE (diluted 1:1000) for 1 hour at room temperature. After washing, the membranes were incubated with 1 mg/mL of streptavidin-peroxidase (Sigma, Poole, United Kingdom) diluted 1:1000 for 1 hour at room temperature. After a final wash, the color was developed with the addition of 1-component 3,3',5,5'-tetramethylbenzidine membrane peroxidase substrate (KPL, Gaithersburg, MD). The color reaction was stopped by washing the membranes with distilled water.

### Gel Electrophoresis Protein Profile Analysis

The Gel Electrophoresis Protein Profile Analysis (GEPPA) program (custom, in-house validated software) is used as a numerical comparator that summarizes the overall IgE reactive similarity between 2 samples run on sodium dodecyl sulfate-polyacrylamide gel electrophoresis (SDS-PAGE) and/or Western blotted with a similarity measure to enable quantitative analysis of gel-based methods. Briefly, an image of a Western blot or SDS-PAGE gel is captured via the Genegenius (Syngene, Cambridge, United Kingdom) and imported into The Genetools program. Genetools program identifies the bands in each lane of the image and, via comparison with molecular weight markers, assigns a molecular weight, band density, and peak height to each band within each lane. This information is fed into the GEPPA program, and based on band matching via molecular weight, peak height, and band density, a similarity measure is generated between the 2 samples. The use of this method to analyze gelbased assays removes bias and subjectivity from what has traditionally been a qualitative method. The program has been validated to demonstrate robust and repeatable analysis of data gathered from the Genetools software via use of calibrated molecular weight markers of known size and mobility.

### IgE and IgG Enzyme-Linked Immunosorbent Assay Potency Determination

Potency enzyme-linked immunosorbent assays (ELISAs) were performed to measure the IgE and IgG reactivity of the grass pollen extracts. IgE reactivity was determined by competing solid-phase grass pollen extract with soluble samples for IgE antibodies. Briefly, microtitre plates (Corning) were incubated overnight at 2 to 8°C with a freeze-dried in-house reference preparation diluted in DPBS containing magnesium chloride and calcium chloride. The plates were washed with DPBS-Tween 20 and blocked with 1% bovine serum albumin solution (Sigma) in coating buffer. After washing, samples were then loaded, followed by human anti-grass IgE sera (pool of 8 patients as detailed above), and incubated for 2 hours with continuous shaking at 20°C. The plates were washed once again and incubated with goat anti-human IgE horseradish peroxidase. The color was then developed by adding 3,3',5,5'-tetramethylbenzidine peroxidase substrate (KPL). The reaction was stopped with 1 M orthophosphoric acid (Fisher, Loughborough, United Kingdom), and the plates were read at 450 nm.

IgG reactivity was determined by measuring the group 1 content using an in-house competition ELISA in conjunction with time-resolved fluorescence. Briefly, microtitre plates (Thermo Fisher Scientific, United Kingdom) were coated with 50 μg/mL of staphylococcal protein-A in DPBS. After washing the plate with DPBS-0.1% Tween 20, the wells were incubated with rabbit anti-group 1 serum at 37°C for 1 hour. This was followed by the addition of a mixture of grass pollen extract and Europium-labeled purified group 1 and incubated at 37°C for 1 hour 30 minutes. The plate was washed again and the reaction developed with enhancement solution. The plate was then read using a time-resolved fluorescence spectrometer (Perkin Elmer, Waltham, MA).

### Phl p 5 Major Allergen Determination

Anti-Phl p 5 mAb 1D11 (Indoor Biotechnologies, Charlottesville, VA) diluted 1:1000 in 50 mM carbonate-bicarbonate buffer was coated onto a NUNC Maxisorp plate and incubated overnight at 2 to 8°C. The plate was washed with 0.5% phosphate-buffered saline-Tween and blocked with 1% bovine serum albumin diluted in 0.5% phosphate-buffered saline-Tween for 30 minutes. After washing as before, samples were loaded and incubated for 1 hour. After a further wash, diluted biotinylated anti-Phl p 5 mAb Bo 1 (Indoor Biotechnologies) was added to the plate and incubated for 1 hour. Streptavidin-peroxidase (0.25 mg/mL) diluted 1:1000 was added to the plate and incubated for 30 minutes after washing. A final wash was performed, and the plates were developed by adding 100 μL of 1 mM 2,2'-azino-bis(3-ethylbenzothiazoline-6-sulphonic acid) (ABTS) in 70 mM citrate-phosphate buffer containing H_2_O_2_. The plate was read when the optical density at 405 nm reaches 2.0 to 2.4.

## Results

### In Vitro Analysis of 13 Grasses

#### Protein Content

Thirteen 5% grass pollen extracts were analyzed for total protein content via the Bradford protein assay [21]. Total protein content (Table 1) varied between each species. The range of data runs from 1645.9 μg/mL for Cultivated Rye to 924 μg/mL for Sweet Vernal grass.

**Table 1:** Results of In Vitro Analysis of 13 Grass Pollen Extracts

Sample Type	Protein Content (μg/mL)	IgG Reactivity (QAU/mL)	IgE Reactivity (QAU/mL)	Phl p 5 Reactivity (μg/mL)
Cultivated Rye grass	1645.90	190.10	22.60	114.20
Crested Dogstail grass	1595.30	1963.50	41.50	219.90
Yorkshire Fog grass	1332.60	4935.50	213.90	133.80
Brome grass	1027.10	193.85	20.10	19.66
False Oat grass	1636.70	2230.00	110.00	273.30
Foxtail Meadow grass	995.00	730.35	71.60	37.87
Timothy grass	1189.00	305.05	136.10	161.40
Fescue Meadow grass	1596.90	4785.50	265.10	231.00
Rye grass	1263.70	6412.00	157.20	205.00
Orchard grass	1191.80	1334.50	135.10	215.95
Meadow grass	1013.70	274.50	42.70	32.85
Sweet Vernal grass	924.90	1756.00	12.30	2.45
Bent grass	1251.00	1154.00	62.70	146.20
All 13 grasses mixed (extracted separately)	1368.80	3865.00	186.30	117.60
All 13 grasses mixed (extracted together)	1318.10	3762.50	197.10	120.40

#### IgG Reactivity

Each species of grass demonstrated IgG binding and thus reactivity (Table 1). A much greater degree of variation is evident than was found in the evaluation of protein content. Cultivated Rye returned the lowest IgG potency of 190.10 quality assurance units per milliliter (QAU/mL, an arbitrary in-house unit) while the highest potency was 6412.00 QAU/mL reported by Rye grass. The degree of variation observed is consistent with what would be expected among species that are highly cross-reactive yet contain unique epitopes.

#### IgE Reactivity

Thirteen grass pollen extracts were analyzed via an in-house IgE inhibition ELISA where the large variation in data observed in the IgG analysis is mirrored, albeit at a slightly reduced level (Table 1). Sweet Vernal grass returned the lowest IgE potency with a figure of 12.30 QAU/mL and Fescue Meadow returned the highest with a potency of 265.10 QAU/mL. The variation in IgE is lower than that for IgG, suggesting greater similarity among IgE epitopes in the grass species.

#### Phl p 5 Reactivity

Each grass sample demonstrated the presence of Phl p 5 reactive protein (Table 1). There is a large range in the data as had been observed in the other potency methods. The lowest reactivity was 2.45 μg/mL observed in Sweet Vernal grass and the highest was 273.30 μg/mL in the False Oat grass. Monoclonal antibodies are very specific, and the assays they underpin are typically highly repeatable. As there is similar variation in the Phl p 5 assay and the non monoclonal assays, it could be inferred that the variation observed is true and not due to assay variability.

### Graphical Interpretation of the In Vitro Data

**Figure 1:**
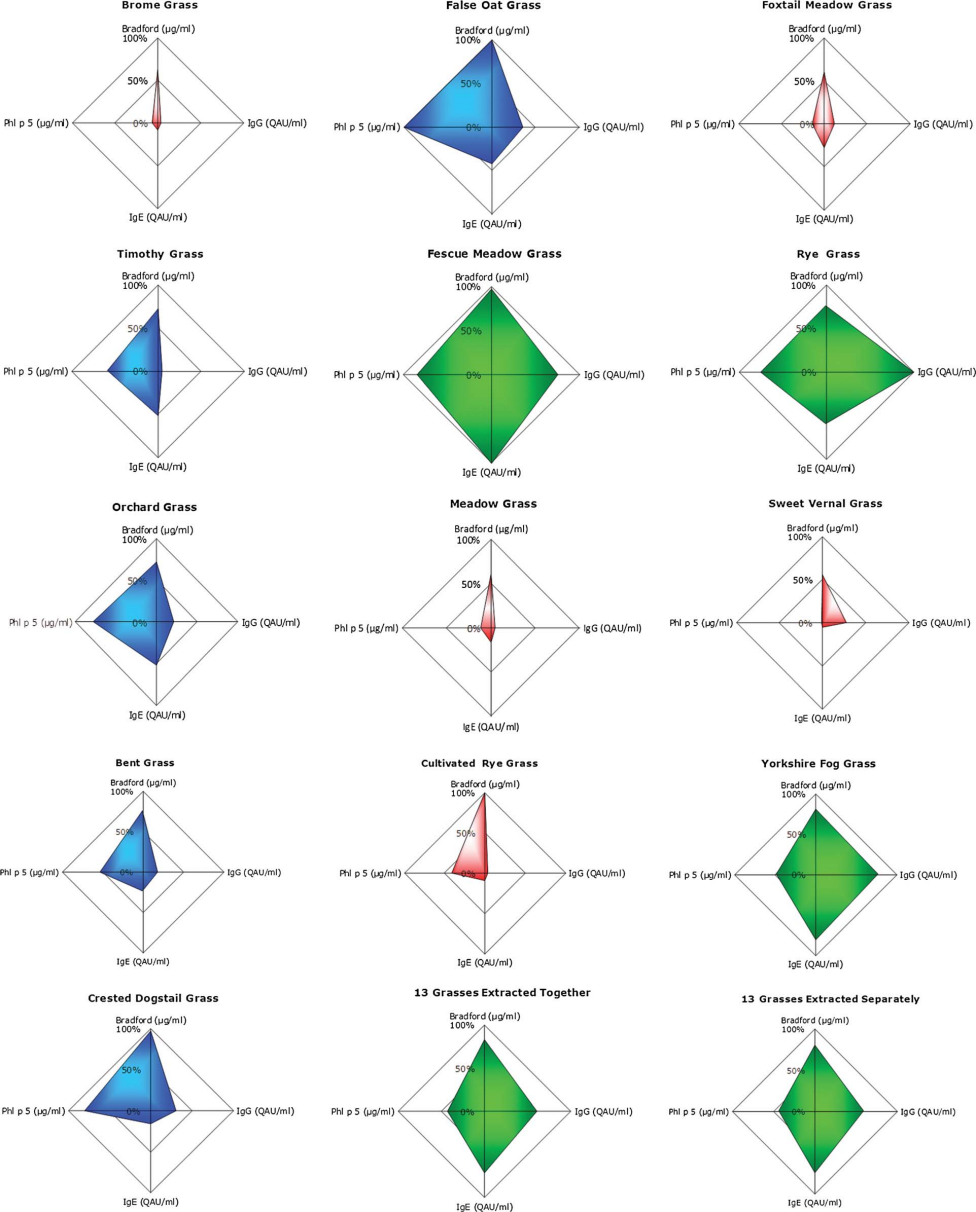
**Radar graphs illustrating the 4 in vitro analysis parameters of the 13 grass species**.

The data in Table 1 have been graphically rendered (Figure 1) using radar graphs to illustrate the differences between each species. Each radar graph is constructed such to present data as a percent of the maximal value of the whole data set. The charts highlight that 3 species (coded green) exhibit the greatest concentration of IgE/IgG reactivity and Phl p 5 reactivity: Fescue Meadow, Rye grass, and Yorkshire Fog grass. Five species are coded blue to indicate a relatively average amount of activity: False Oat, Timothy, Cocksfoot, Bent grass, and Crested Dogstail. The remaining 5 species (coded red) contain the lowest amounts of active protein: Meadow grass, Brome grass, Foxtail Meadow, Sweet Vernal, and Cultivated Rye.

The data (Table 1 and Figure 1) detailing the effect of extracting each of the 13 grasses together and extracting each of the grasses separately and mixing highlight that there is no difference in immunogenic or allergomic content of the mixes.

### GEPPA Analysis of IgE Immunoblots of 13 Grass Species

**Figure 2:**
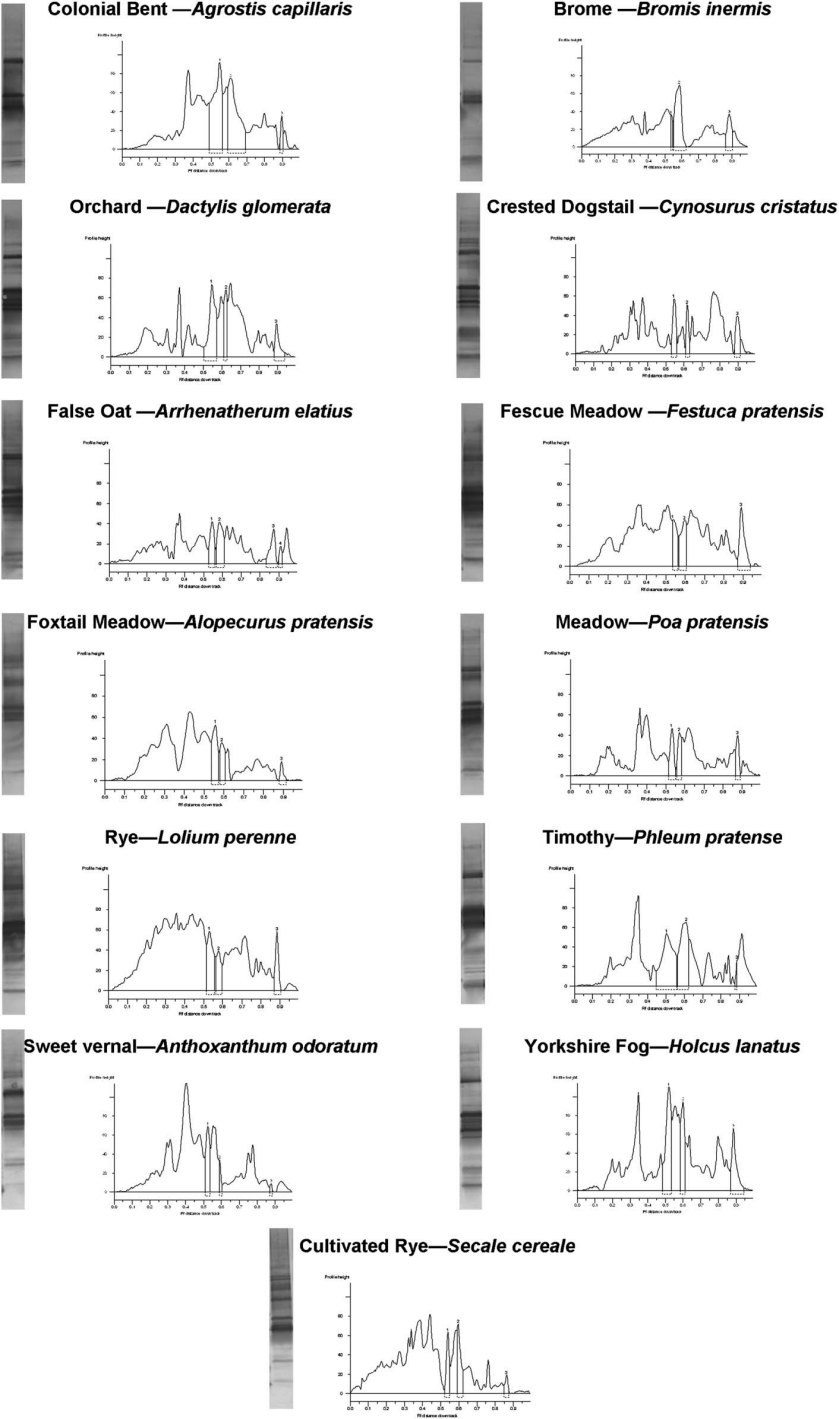
**Illustrating the blots, peaks, and heights of the 13 grass species Western blotted using human IgE serum**.

Western blotting was used to visualize the IgE reactive proteins of each of the 13 grass species. Figure 2 details the identified peaks and height reported by Gene Genius software of each of the grass samples. Using GEPPA to interpret these data, it is possible to quantitatively compare multiple lanes and determine the degree of similarity (Tables 2 and 3).

**Table 2:** GEPPA Analysis Showing the Similarity Measure of Each of the 12 Grasses to the 13th Grass--Crested Dogstail

Primary Sample	Secondary Sample	Similarity Measure (%)	Difference From Mean (δ)
Crested Dogstail	Colonial Bent	66.80	+3.08
Crested Dogstail	Brome	58.40	-5.32
Crested Dogstail	Orchard	72.10	+8.38
Crested Dogstail	False Oat	65.80	+2.08
Crested Dogstail	Fescue Meadow	59.10	-4.62
Crested Dogstail	Foxtail Meadow	61.70	-2.02
Crested Dogstail	Meadow	65.00	+1.28
Crested Dogstail	Rye Grass	55.90	-7.82
Crested Dogstail	Timothy	60.70	-3.02
Crested Dogstail	Sweet Vernal	66.10	+2.38
Crested Dogstail	Yorkshire Fog	67.70	+3.98
Crested Dogstail	Cultivated Rye	65.30	+1.58
Similarity mean	--	63.72	--

**Table 3:** GEPPA Analysis Showing the Similarity Measure of Each of the 12 Grasses to the 13th (Exemplar) Grass--Timothy

Primary Sample	Secondary Sample	Similarity Measure (%)	Difference From Mean (δ)
Timothy	Colonial Bent	77.70	+10.39
Timothy	Brome	82.50	+15.19
Timothy	Orchard	62.20	-5.11
Timothy	False Oat	62.80	-4.51
Timothy	Fescue Meadow	77.40	+10.09
Timothy	Foxtail Meadow	57.80	-9.51
Timothy	Meadow	72.30	+4.99
Timothy	Rye Grass	68.50	+1.19
Timothy	Crested Dogstail	60.70	-6.61
Timothy	Sweet Vernal	39.80	-27.51
Timothy	Yorkshire Fog	82.20	+14.89
Timothy	Cultivated Rye	63.80	-3.51
Similarity Mean	--	67.31	--

The calculated molecular weight of each band along with band density and height are used by GEPPA to calculate a numeral similarity measure useful in providing a quantitative comparison of 2 samples. Each of the samples evaluated by Western blotting were analyzed (Table 2) as a comparison of the profile of Crested Dogstail against the other 12 grass species.

There is significant homology between the Crested Dogstail grass and the other 12 grass species (Table 2). Each grass exhibits a greater than 55% similarity measure, which indicates that a significant number of allergenic proteins have been found to match. However, these data also indicate that there are minor differences between species possibly attributable to unique epitopes not shared among the species. The mean similarity is 63.7%, and the variation is δ = +8.38 to -7.82 from the mean.

Table 3 details the GEPPA comparison when Timothy grass is used as the comparator. The data broadly mirror those seen in Table 2 where there is demonstrated homology and similarity between the grass species, which is supportive of grouping. However, Sweet Vernal grass is only 39.8% similar to Timothy grass (even though they are considered homologous [18]), whereas it is 66.1% similar to Crested Dogstail (Table 2). The mean similarity of 67.31% corresponds to that found in Table 2; however, the variation is δ = +15.19 to -27.51, which is considerably more variable than the Crested Dogstail comparison.

### Epitope Specificity

A number of Western blots of each of the 13 grass species (Table 4) were run to evaluate epitope specificity and sharing using different sera pools. Human anti-grass IgE serum was pooled with 5% Timothy native extract to block Timothy grass epitopes and was used to probe one of the gels.

**Table 4:** Identity of Grass Species in Each Lane in Western Blots (Figures 3-5)

Lane Number	Sample Identity
1	Molecular weight marker
2	Foxtail
3	Crested Dogstail
4	Sweet Vernal
5	Brome
6	Orchard
7	Fescue Meadow
8	Meadow
9	Timothy
10	Rye Grass
11	Cultivated Rye
12	False Oat
13	Bent
14	Yorkshire Fog
15	Molecular weight marker
16	Molecular weight marker

**Figure 3:**
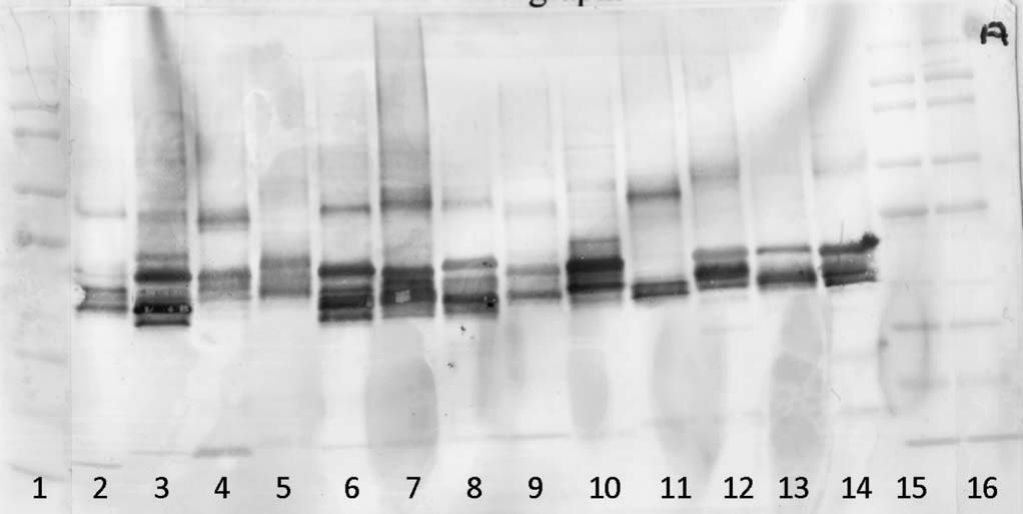
**Demonstrating the IgE reactivity of each of the 13 grass species using a serum pool comprising 8 grass allergic patients**.

**Figure 4:**
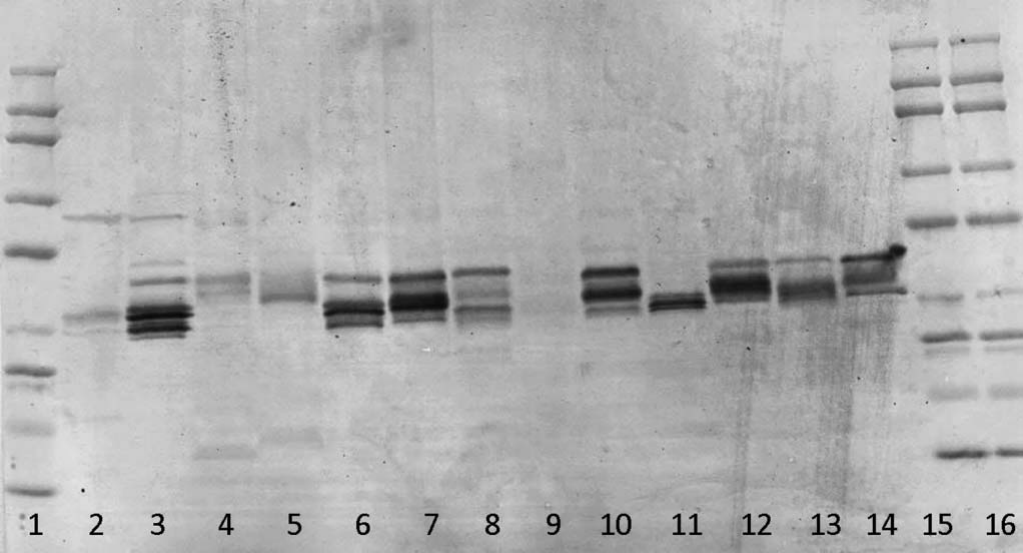
**Demonstrating the IgE reactivity of each of the 13 grass species using a serum pool comprising 20 grass allergic patients with all Timothy grass epitopes blocked**.

Figure 3 illustrates the Western blot probed with sera where there are no blocked epitopes (pool of 8 patients). As has been previously demonstrated [22], there is clear evidence of similar reactivity among the grasses, yet subtle differences are evident illustrating the unique allergenic attributes previously discussed. The use of a serum pool blocked to Timothy epitopes (Figure 4) reveals unique epitopes present in each grass species. There is no banding present in the lane containing Timothy grass, demonstrating that the blocking was effective, yet there is clear banding in each other grass species, indicating IgE reactivity to epitopes not present in Timothy grass.

**Figure 5:**
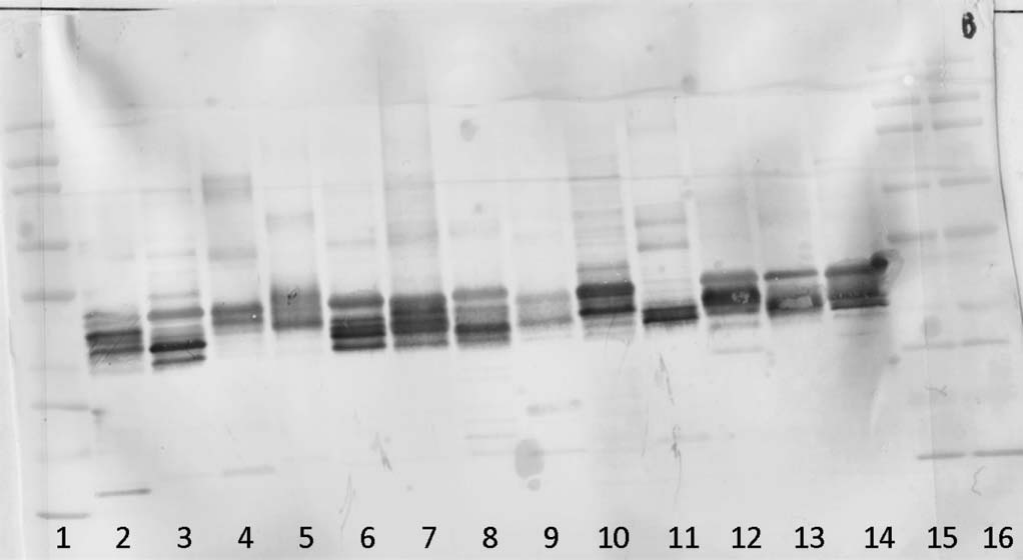
**Demonstrating the IgE reactivity of each of the 13 grass species using a serum pool comprising 20 grass allergic patients**.

Finally, Figure 5 illustrates the Western blot where a serum pool composed of 20 grass allergic patients was used. Epitope recognition, band density, and specificity are indistinguishable from those present in Figure 3, where 8 patients made up the pool.

## Discussion

It is proposed that, due in part to the varied species geography, exposure conditions, and patient diversity, no immunotherapeutic can treat all patients. Moingeon et al [19] indicate that an immunotherapy consisting of multiple species better reflects natural exposure conditions, and in work published in 2011, Bullimore et al [22] noted the same to be likely.

Grass pollens from 13 different species were extracted under identical conditions and yielded different profiles in terms of protein content, IgG, IgE, and Phl p 5 reactivity. Sweet Vernal grass (*Anthoxanthum odoratum*) was found to contain the least amount of protein of all the grasses tested, the least amount of Phl p 5 major allergen reactivity, and comparatively low levels of IgE and IgG reactive protein. Conversely, Cultivated Rye (*Secale cereale*) was discovered to contain the highest recorded amount of total protein, however exhibits similar low levels of IgG and IgE reactive protein as that found in Sweet Vernal grass. These data indicate that the differences in biological character are not attributable to the ability to extract protein from the raw material but rather from inherent differences between each of the grasses.

No single grass species examined displays an identical biological profile (Figure 1); of particular interest are the data recorded for the considered exemplar grass of the Pooideae family, Timothy. The data indicate comparatively lower content for protein, lower IgE and Phl p 5 reactivity, and there is only a fraction of the IgG reactivity found in the other grass species analyzed. A vigorous IgG response is implicated in effective SIT [10], and these observations suggest that in therapies consisting of only single species, the potential lack of sufficient IgG reactivity could impact effectiveness.

The differences in immunogenicity between each of the 5% (w/v) grass extracts are not directly comparable with studies conducted on purified allergens nor samples where the amount of allergen has been levelled across species. The low IgG reactivity observed in Timothy grass compared with the other species does not imply that Timothy is weakly IgG reactive; merely that for a given amount of raw material, there is less IgG reactive protein per gram. If some grass species contain more immunogenic material per unit volume as these data suggest, then it is possible to infer that patients exposed to pollen from these species would, by default, be exposed to more immunogenic material too.

The relative differences in IgE reactivity of each of the analyzed grass species is interesting. Of the 13 grasses analyzed, only Rye grass and Yorkshire Fog exhibit > 50% of the IgE reactivity of the grass with the most IgE reactivity, Fescue Meadow grass. The remaining 10 species (including the considered exemplar Timothy grass) possess ≤ 50% of the IgE reactivity. IgE epitopes are recognized as the causative agent of allergy; the relative differences seen in IgE reactivity compared with protein content could be indicative of a propensity for more/less aggressive sensitization profiles. Should a therapy be designed based on targeting a wide population of sensitization profiles, it may be wise to consider immunotherapy with multiple grass species.

This is corroborated by Figure 4 illustrating that the use of sera with Timothy-specific epitopes blocked shows significant reactivity to each of the other grass species studied. This is indicative of unique epitopes present in the other grass species that are not present in Timothy grass and not solely indicative of cross-reactivity as speculated previously. Cross-reactivity is no doubt present; however, Figure 4 suggests that SIT using only Timothy grass may not treat all grass-sensitive patients.

The in vitro analysis of 13 grass species indicates that the use of multiple species could negate the need to include high quantities of raw material to compensate for low yield (which could potentially lead to high levels of nonreactive material or complicated purification methods) while maintaining allergenic and cross-species reactivity.

Our present data can be used to highlight the importance of appropriate allergen mixes in diagnosis and in immunotherapy. Diagnosis of allergic sensitization is commonly performed using skin prick testing. This, together with the case history, is maintained as the current practical method to determine suitability for SIT. The data herein regarding the unique allergenic properties of certain grass species, substantiated by the description by Moingeon et al [19] regarding natural exposure conditions, suggest that allergen mixes could offer advantages to diagnosis. Diagnosis using recombinant allergens or a single species may lead to some patients sensitized to minor allergens or alternate grasses being missed. However, a diagnostic test comprised the 13 grass species tested in this investigation could further cover the potential patient population and result in fewer missed diagnoses.

GEPPA is a technique whereby the determined molecular weight, band density, and peak height of separated proteins are used to calculate a "similarity measure." The similarity measure is a quantitative means for the interpretation of SDS-PAGE/Western blot profiles. The development of GEPPA was initiated out of a need for a repeatable nonbiased method to compare different samples run using SDS-PAGE and/or Western blotting. Conventional methods can be influenced by visual subjectivity and interpretation, so a method that reduces this apparent bias would be advantageous. GEPPA is a validated technique that has been used to enable instant comparison and reproducibility of reference material compared with test samples and has been used in the present study to compare the similarity of each of 13 grass species. Western blots were generated using a pool of human patient grass-positive sera.

Data indicate that there is a high degree of homology, and Crested Dogstail is considered similar to each of the other 12 species (Table 2). Eight of the 12 grasses analyzed via GEPPA against Crested Dogstail exhibited a similarity measure of > 60% with the remaining 5 demonstrating a > 55% similarity measure. The similarity measure of Crested Dogstail compared with Timothy is also comparable with the other grass species (Table 3). Here, all species demonstrate a similarity measure of > 58% apart from Sweet Vernal grass (38%).

Aside from Crested Dogstail, each grass is considered grouped; justification for the inclusion of Crested Dogstail into the sweet grasses group of the Pooideae family as defined by Lorenz et al [18] is evident because of the close correlation of the similarity measure of Crested Dogstail to the already grouped species (Tables 2 and 3). The levels of similarity can only be possible because of analogous molecular profile and extensive sharing of epitopes among the grass group, which in itself could be considered justification for the inclusion of Crested Dogstail to the sweet grasses group.

These findings are corroborated by the work by Bullimore et al [22], where homology between each of the 13 grass species used within a 13-grass immunotherapeutic via mass spectrometry was demonstrated. The data established structural homology and epitope sharing between species consistent with criteria Lorenz et al [18], detail to be sufficient to enable the inclusion of crested dogstail within the sweet grasses group of the Pooideae family.

Our findings illustrate that there is greater variation in similarity measure when Timothy grass is used as the exemplar grass species compared with when Crested Dogstail is used (Tables 2 and 3, respectively). This correlates with the in vitro data presented in this study, which opens questions as to whether the current strategy for dealing with data extrapolation from one species to another in terms of homologous groups is appropriate.

Lorenz et al [18] suggest that there are 2 grass species that may act as the representative allergen source for the homologous group of sweet grasses of the Poaceae (Gramineae) family (including those listed with reservations): Timothy or Kentucky Bluegrass. We propose that, because of the homology demonstrated by Lorenz et al [18] in their proposal and backed by our own findings [22], the principles of homologous groups regarding the sweet grasses of the Poaceae (Gramineae) family be maintained albeit with the addition of Crested Dogstail (*C. cristatus*) to the group.

However, we suggest that any member of the group could act as the exemplar species based on biochemical assessment, not just Timothy grass or Kentucky Bluegrass. There is no biochemical justification as to why it is acceptable to infer data from Timothy grass on to Sweet Vernal grass but not the other way round. In our suggested model of homologous grouping, if species are determined to be homologous, then any species can be the exemplar.

## Competing interests

The authors declare that they have no competing interests.
